# Perception of climate change effects on water resources: Iraqi undergraduates as a case study

**DOI:** 10.1007/s12517-022-09695-y

**Published:** 2022-03-10

**Authors:** Laheab A. Al-Maliki, Sohaib K. Al-Mamoori, Ihsan A. Jasim, Khaled El-Tawel, Nadhir Al-Ansari, Fadi G. Comair

**Affiliations:** 1 Department of Regional Planning, Faculty of Physical Planning, University of Kufa, Najaf, Iraq; 2 Department of Environmental Planning, Faculty of Physical Planning, University of Kufa, Najaf, Iraq; 3 Department of Architecture Engineering, Wasit University, Kut, Iraq; 4 Faculty of Engineering, Lebanese University, Beirut, Lebanon; 5 Department of Civil, Environmental and Natural Resources Engineering, Lulea University of Technology, Lulea, Sweden; 6 UNESCO IHP Council, Paris, France

**Keywords:** Questionnaires analysis, Climate change mitigations, Academic awareness, University students, Water resources

## Abstract

Developing countries suffer from the effects of climate change on water resources more than other countries. This paper aims to specify the undergraduates’ knowledge about climate change effects on water resources. This study was conducted through a questionnaire distributed using Google form in May 2021. Descriptive analysis was used to display the level of awareness, and then the correlation between the respondents’ gender, stage, and scientific specialization were analyzed. The results showed that the general knowledge for all questionaries ranged between 40 and 50 %. Moreover, a weak positive correlation with the highest Spearman’s rho of 0.15 was shown for the students’ gender with their understanding of climate change main cause by 0.06 for the academic stage versus knowledge of climate change causes. Moreover, the results indicated a weak role for university education in exchange for a prominent role for television and social media in raising awareness. The research recommends integrating the Environmental Education (EE) programs into the Iraqi educational systems as it proposes a systematic educational method through which climate change and other environmental problems may be addressed holistically at all educational levels.

## Introduction

Climate change and the associated global warming are among the most severe problems of the era (Botzen et al. 2021; Kumar et al. 2021; Amin et al. 2016). Many severe and possibly permanent changes in our planet’s ecological and geological systems are consequences of climate change (Hoegh-Guldberg and Bruno 2010; Buytaert et al. 2011; Brierley and Kingsford 2009; Santos et al. 2015). Climate change and global warming are often confused and interchangeably (Whitmarsh 2008; Giwa et al. 2021). Climate change can be defined as changes in the atmosphere layers, such as temperature, precipitation, and other climate variables. Global warming is one of the consequences of climate change, related to the rise in the average temperature near the Earth’s surface. Climate change happens for two reasons: the natural causes which happened at long-term period and the causes related to human activities. According to the International Panel on Climate Change (IPCC), most heat observed over the last 50 years can be attributed to human activities(McMichael 2003; Nesmith et al. 2021; Besley and Peters 2020). These changes have caused many environmental problems affecting human health; some are the depletion of the ozone layer, the spread of infectious diseases, pressures on natural resources, and freshwater scarcity (Organization 2008; McMichael et al. 2003; Norval et al. 2007; Kinney 2018). Many scholars have studied the future climate projections model, and they figured out that climate change is happening and its impact on water resources is inevitable (Kumar et al. 2021; Salimi et al. 2021; Konapala et al. 2020).

Climate variability and change influences ground and surface water systems both directly through replenishment by recharge and evaporation and indirectly through changes in water use (Taylor et al. 2013; Kløve et al. 2014; Kareem et al. 2021). The catastrophic impacts of climate change on water resources include uneven precipitation distribution temporally and spatially (Graef and Haigis 2001; Shawul and Chakma 2020; Mandal et al. 2021), decreasing snow cover resulting from warmer temperatures, floods, drought, and sea-level rise (Adamo et al. 2018; Das and Goyal 2021; Kareem et al. 2020) and rising groundwater levels. The weakness and fragility of water management systems in Iraq, in addition to its arid and warmer climate, and its increased exposure to extreme weather events, increase its vulnerability to climate change more than other countries (Carattini et al. 2020; Wynes and Nicholas 2017; Mohammad 2014). It is impossible to address climate change and bring about a fundamental change in reducing its environmental effects if it is kept within the ambit of the scientific elite and confined to academic frameworks. Real change requires that society adopts confronting the impacts of climate change. This, in turn, requires spreading awareness about the current and future causes and impacts of climate change so that there is a real value for national plans and international adaptation and mitigation initiatives.

Recent research on public perceptions of climate change has improved our understanding of the lay public’s evolving response, as the levels of climate change awareness, knowledge, perceived risk, and support for mitigation or adaptation vary significantly across the world (Xie et al. 2019; Lee et al. 2015; Yomo et al. 2020). However, strengthening adaptive capacities in developing countries needs to focus on promoting these measures at all levels (Sun and Han 2018). Thus, for societies to prepare and educate for climate change adaptation initiatives, one of the most important rules is to know the public and design the education program according to their preconditions to facilitate the achievement of the change effects through the participation and support of the people (Paerregaard 2020; Kuthe et al. 2019; Luís et al. 2018).

The term climate change awareness summarizes the factors that describe and determine the participation of people in creating an environmentally and climate-friendly society (Kuthe et al. 2019; Mermer 2010; Cannon et al. 2020); as to understand the environmental issues better, it is essential to adjust the social understandings and actions to support the changing climatic conditions (Craig et al. 2019; Jasim et al. 2021). This research tries to investigate the university students’ awareness about climate change, for they are considered the leaders of the future and the conscious and influential class in society. Knowing the extent to which university students are aware of this vital topic will be the basis for starting towards change in the way we spread knowledge and integrating society with these environmental issues through various programs if there is a lack of awareness. However, if the students’ understanding is good, it is possible to apply climate change adaptation and mitigation measures.

Although many studies assess awareness of climate change, Iraq has not been included in these studies due to incomplete national development and characteristic vulnerability data. However, since Iraq launched a national adaption plan in September 2020, it has become essential to support these efforts. This study was conducted through a questionnaire distributed using Google form and targeted Iraq under graduated students to assess their knowledge about climate change effects on water resources. The results of this study will be helpful for researchers and decision-makers in Iraq to identify weaknesses in students’ awareness and form a perception about the best ways to disseminate information aimed at the success of adaptation and mitigation initiatives.

## Materials and methods

### Climate change concept

#### Climate change causes

The Earth’s climate has undergone many changes in the past over millions of years (Hegerl et al. 2019). One of the natural causes that caused long-term climate change is volcanic activity, which releases large amounts of carbon dioxide into the atmosphere in addition to the amount of solar energy that reaches the earth surface. These changes have resulted in enormous environmental changes that affect the ecosystem and human civilizations in many ways (Freeman et al. 2018; Freije et al. 2017).

**Fig. 1:**
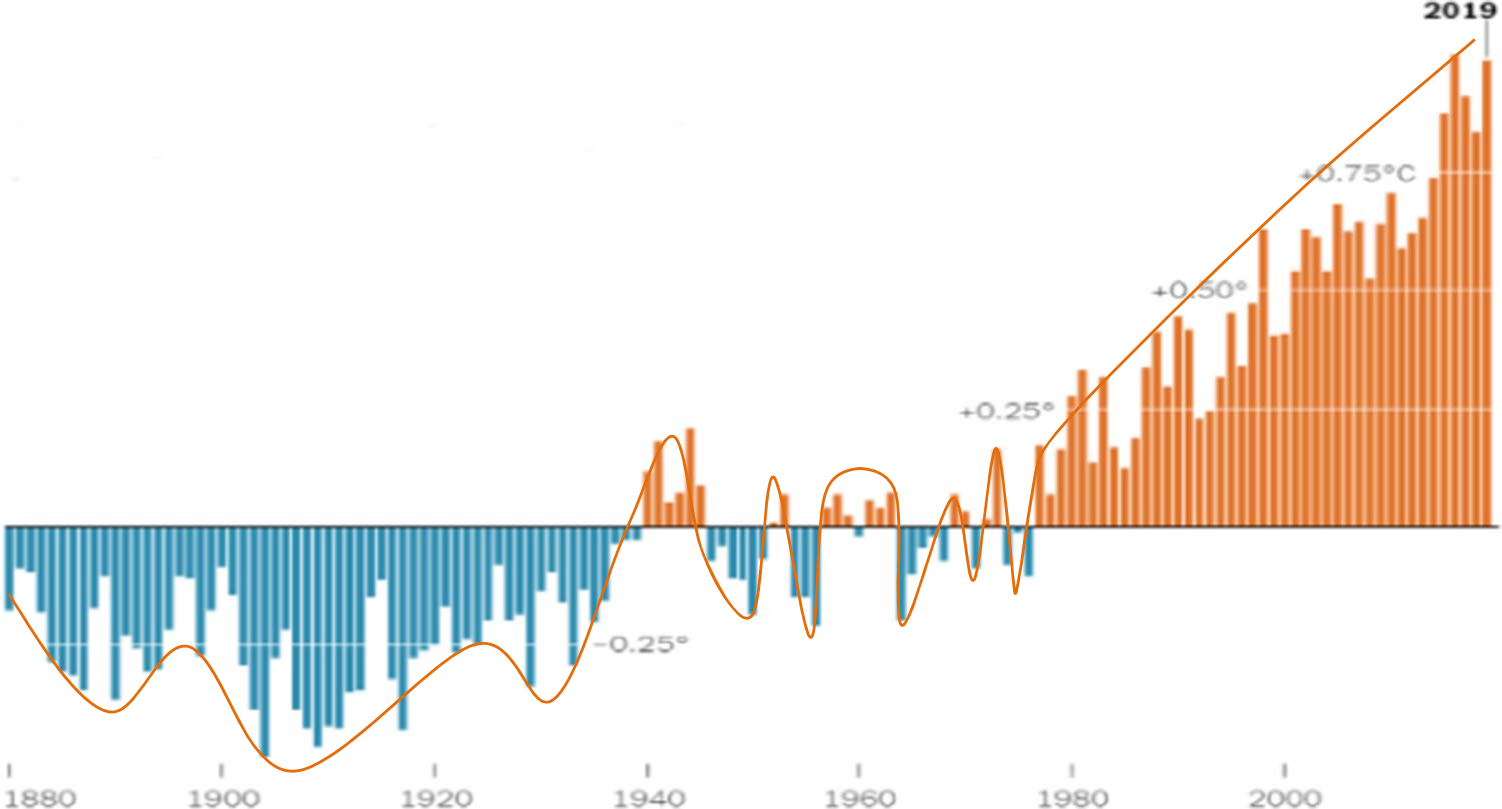
The global average temperatures compared to the middle of the twentieth century

Although the climate changes naturally over many years, studies and research confirm a rapid change in climate due to human activity (Druckman and McGrath 2019; Zscheischler et al. 2018). This apparent change has accompanied the industrial and technological development that has accelerated since the middle of the last century (Khairullina et al. 2019) (Fig. 1). The industrial activities upon which the new nature of life depends has dramatically increased the levels of greenhouse gases — such as carbon dioxide, methane, and nitrogen oxide —in the atmosphere (Al-Ghussain 2019; Kweku et al. 2017).

In addition to the industrial gases emissions, forest harvesting causes the carbon stored in the soil to be released into the atmosphere, thus increasing climate change (Prevedello et al. 2019). It is noteworthy that the quantities of CO2 stored in the soil are about 2000 billion tons (GT) (Cerri et al. 2018; Lejeune et al. 2018; Iordan et al. 2018).

Another reason for the greenhouse gas emission increase is burning fossil fuels to produce energy that covers 80% of global energy demands (Letcher 2020). Fossil fuels are extracted from fossil materials such as coal, natural gas, and oil. These materials, in turn, are extracted and burned to produce heat that is used in all fields.

#### Climate change effects

**Fig. 2:**
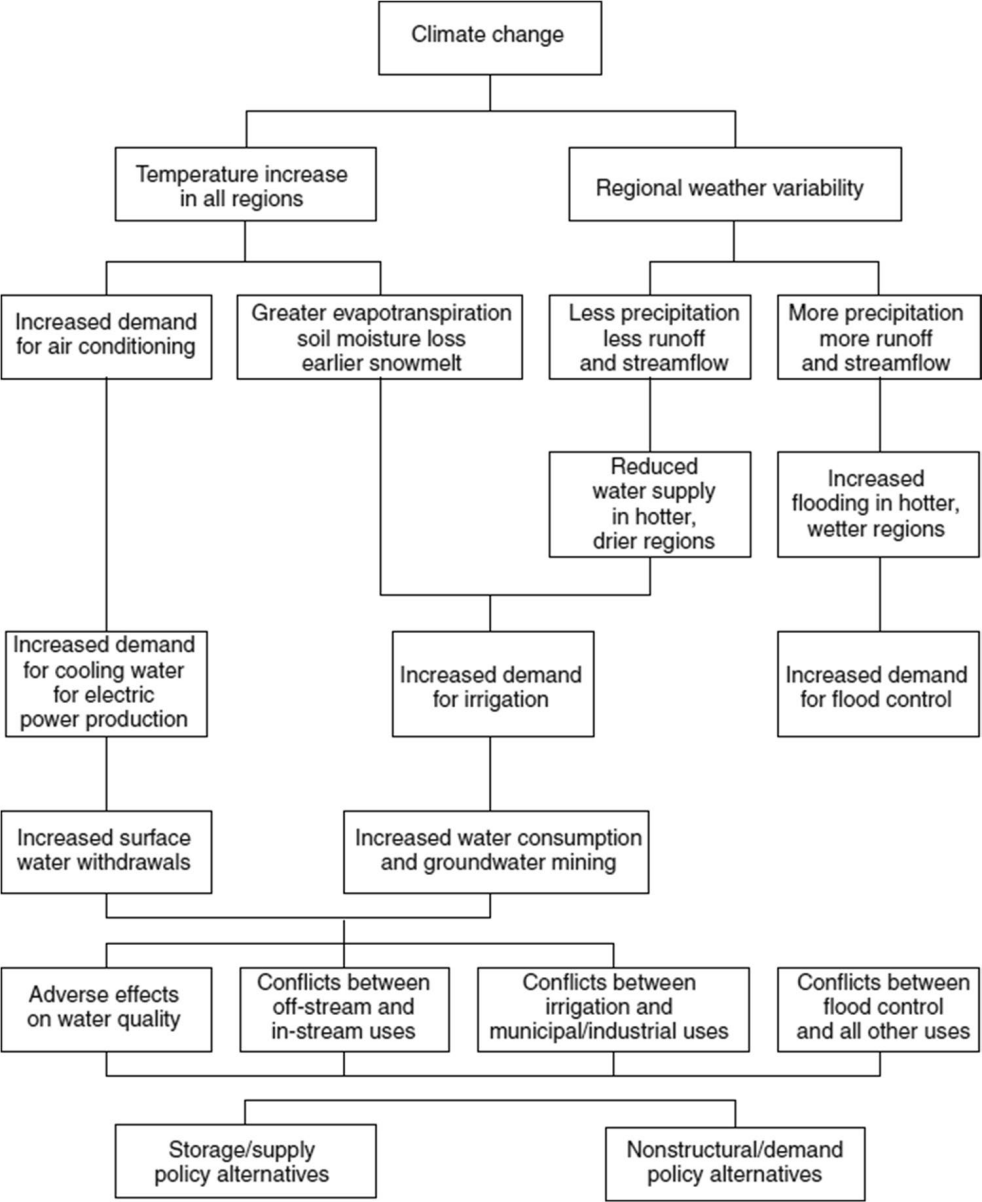
Impacts of climate change on water supply and demand (Hardy 2003)

Climate change can cause significant impacts on water resources and the hydrological cycle. It resulted in the fast melting of the North and South Pole’s snowpack and the associated sea level rising; changes of the precipitation pattern over almost all parts of the world can also be mentioned. This, in turn, affects water supply and demand (Fig. 2). The main effects of climate change are as follows:

##### *Snow and ice melting*

The high temperatures caused the ice sheet to decrease. Studies and research proved that the Arctic ice cover decreased by 10% during the period (1975–1995) (Al-Maliki et al. 2021)

In addition, the snow melting began to happen earlier in recent years than it was in the 1940s of the last century. This melting causes many environmental hazards such as sea-level rise, increased precipitation in certain areas, and the resulting spatial shifts in storm tracks (Luo et al. 2019).

##### *Sea-level rise*

The sea level is rising at an unprecedented rate. As the water warms, its volume expands. In the case of the ocean, this can only result in a rise in sea level relative to the land. About two-thirds of the twentieth-century sea-level rise results from thermal expansion of ocean water and one-third from melting glaciers and ice caps that add fresh water to the sea (Fu et al. 2013; Al-Mamoori and Al-Maliki 2016).

##### *Drought*

Drought is a recurring extreme climate event over land characterized by below-normal precipitation and often associated with warm temperatures over months to years (Williams et al. 2015; Dai 2011; Fernando et al. 2019).

#### Climate change mitigation measures

Some recent studies argue that increased water use efficiency by plants under elevated CO2 may reduce the evaporative demand and therefore mitigate the drying (Dai et al. 2018).

##### Study design

This study was conducted to examine the climate awareness of university students in various scientific disciplines and academic stages.

The questionnaire was designed and distributed by a Google form in May 2021, and it needs 5 min or less to be completed. The questionnaire targeted undergraduate students (average age between 18 and 22 years) in some Iraqi universities. The participated universities are Central Technical University, AlQasim Green University, Al-kafeel University, University of Kufa, Babylon University, University of Wasit, Karbala University, Basra University, and Tikrit University.

The enrolled student number was (*N* = 1052) in which the majority of them were females (592).

The surveys were distributed via email. All students have a verified university email address to be used in their lectures because of the complete and partial ban resulting from the coronavirus pandemic conditions, which helped obtain an appropriate sample for the study.

#### Sample size calculations

According to the Central Statistical Organization (CSO), Iraq, the total number of post-graduation students was 1093687. Steven K. Thompson equation was used to calculate the sample size as follows:

1 $$\boldsymbol{n}=\frac{\boldsymbol{N}\ \boldsymbol{p}\ \left(\mathbf{1}-\boldsymbol{p}\right)}{\left(\boldsymbol{N}-\mathbf{1}\right)\left(\frac{{\boldsymbol{d}}^{\mathbf{2}}}{{\boldsymbol{z}}^{\mathbf{2}}}\right)+\boldsymbol{p}\left(\mathbf{1}-\boldsymbol{p}\right)}$$

Where

*n*: Sample Size (

*N*: Population size (1093687)

*z*: Confidence level at 95% (1.96)

*d*: Error proportion (0.05)

*p*: probability (50%)

The resulting sample size was 385, and the respondents’ number in this study was 1052.

#### Questionnaires analysis

When studying climate change-induced disasters or crises, Hein et al. (2019) noted that “the use of natural experimental approach can be used for precise estimation of disaster impact” (Hein et al. 2019; Luke 2002). The statistical analysis was performed using the Statistical Package for Social Science (SPSS 20 for windows) and statistical package from Excel 2019 (Microsoft Corporation) for comparison of means.

A reliability check was carried out using Cronbach’s alpha to indicate the validity of the questions before they were distributed. Its value was 0.772 (Table 1), which means the stability of the questionnaires.

**Table 1:** Reliability statistics

**Cronbach’s alpha**	***N*** **of Items**
0.772	9

#### Respondents’ background

To determine the students’ awareness level and the higher education contribution in spreading this awareness, the scientific disciplines were classified according to Table 2. This classification aims to find out which fields need more support concerning climate change and which have preliminary information on the subject. It is worth noting that the institutes are classified in this study under the category “Others,” and the student graduates after 2 years of study. In general medicine specialization, the student graduates after six study stages, while in dentistry and pharmacy, the student studies for 5 academic years. In the rest of the disciplines, the student graduates after 4 academic years.

**Table 2:** The respondents’ statistics

Category		Number of respondents	Percentage %
**Scientific specialization**	Medical sciences	131	12
	Engineering sciences	392	37
	Applied and pure sciences	79	8
	Agriculture and veterinary	37	4
	Human Studies	116	11
	Literary disciplines	84	8
	Artistic disciplines	3	0
	Others	210	20
**Total**		1052	100
**University stage**	First stage	279	26.5
	Second stage	270	25.7
	Third stage	189	18.0
	Fourth stage	204	19.4
	Fifth	36	3.4
	Sixth stage	74	7.0
**Total**		1052	100

#### Questionnaires administration

Questionaries were developed to quantify the university student’s knowledge of climate change causes and effects. The questionaries were divided into three axes: the causes, the effects, and the mitigation measures of climate change (Table 3).

**Table 3:** The questionaries

**Believing in climate change**	Climate change is evident in extreme weather events, and it is already happening
**Climate change definition**	The emission of gases into the atmosphere that causes global warming Changes that occur in the layers of the atmosphere, such as temperature and precipitation Excessive emission of gases Studying the human impact on the environment
**The source of prior knowledge about climate change**	University course Television Social Media
**Climate change causes**	Human Activity (Burning fossil fuels, increasing the population, increasing industrial activity and the number of cars and gases emitted) Natural Causes (Volcanic eruption and increase in solar activity) Both Human Activity and Natural Causes
**Climate change effects**	Climate change is evident in extreme weather events, and it is actually happening
	Climate change causes floods
	Climate change causes droughts
	Climate change causes less rain and higher temperatures
	Climate change is causing sea-level rise
	Climate change negatively affects the amount of water in rivers and groundwater
	Climate change negatively affects the quality of river water and groundwater
**Climate change mitigation**	The use of modern irrigation techniques reduces the impact of climate change on water resources
	Increasing awareness of rationalizing water consumption reduces the impact of climate change on water resources

## Results and discussions

### Respondents’ background

**Fig. 3:**
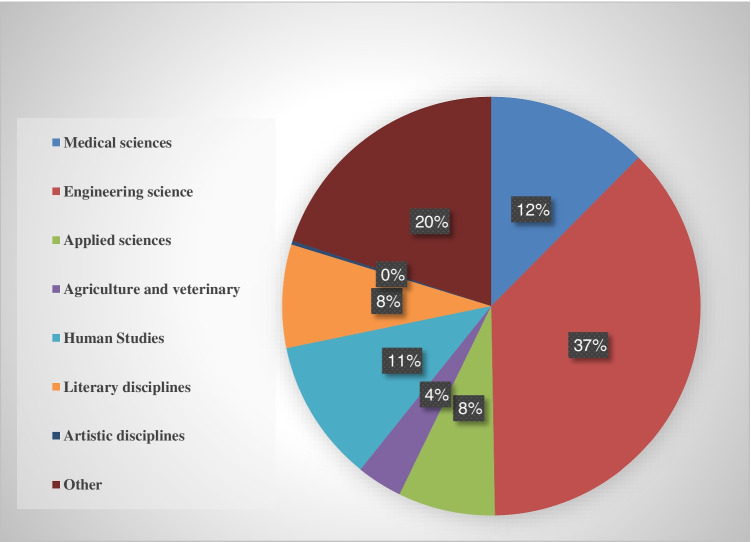
Percentage of respondents’ majors

The largest proportion of students (37 %) studied engineering sciences, while a significant proportion (20 %) studied medical sciences. The least studied fields were the artistic disciplines (0.0%). Figure 3 presents responses by fields.

**Fig. 4:**
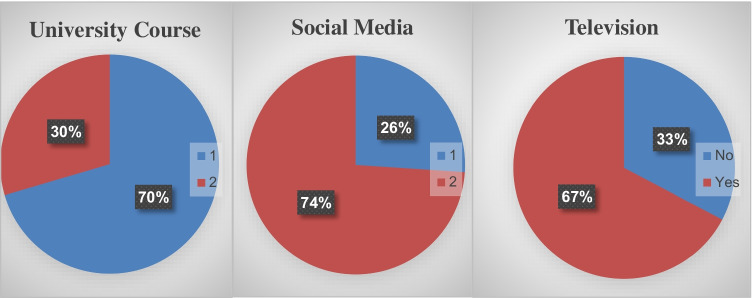
Respondents’ knowledge source regarding climate change

To find out the source of the students’ information on climate change, they were given three options: the university, television, and social media sites. The answers show that the universities role percentage is low (30 %), and this is because there are no courses, whether compulsory or optional, in higher education curricula. Even though some students have acquired this knowledge from university classes, this is due to the reports and extra-curricular activities that include climate change. At the same time, television and social media play an essential role in raising awareness. Table 4 and Figure 4 show the respondents’ information source about climate change.

**Table 4:** Statistics of respondents’ knowledge source regarding climate change

Knowledge source	Answer	Response number	Response percent
University course	No	741	70.4
	Yes	311	29.6
Television	No	344	32.7
	Yes	708	67.3
Social media	No	274	26.0
	Yes	778	74.0

### Respondents’ knowledge of the climate change causes

**Fig. 5:**
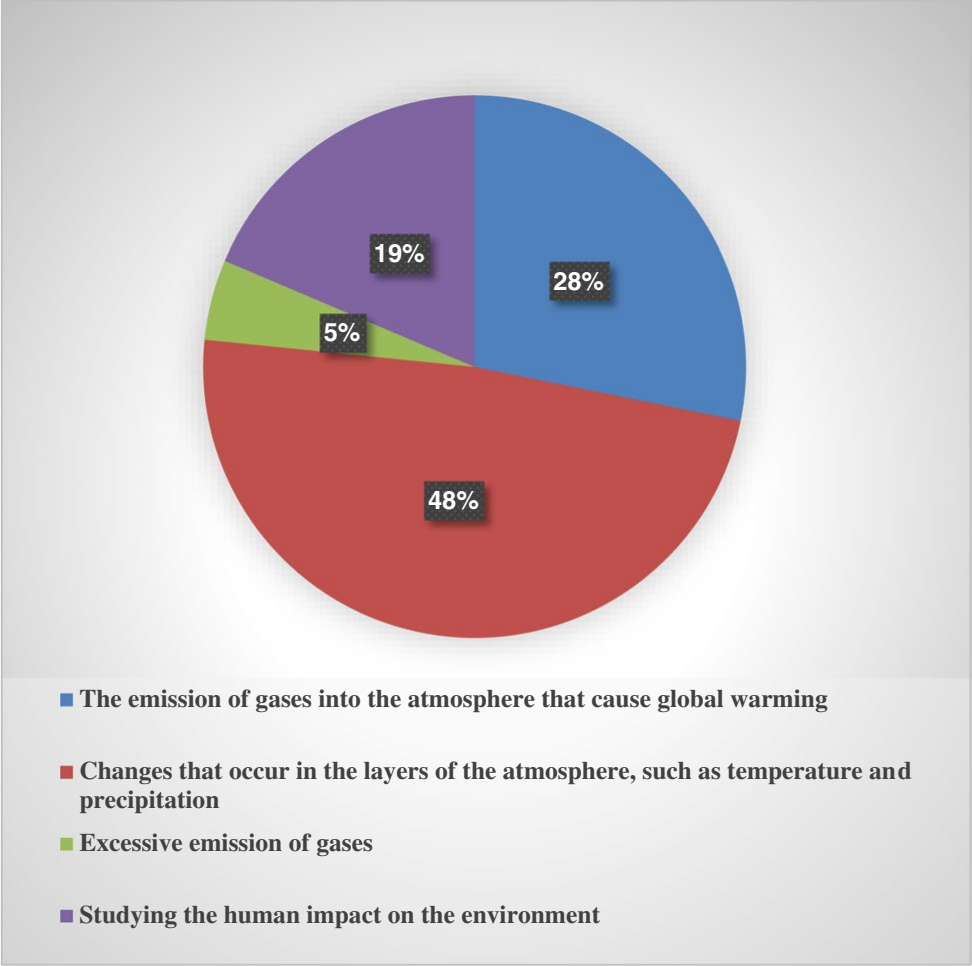
Respondents’ knowledge of climate change definition

**Fig. 6:**
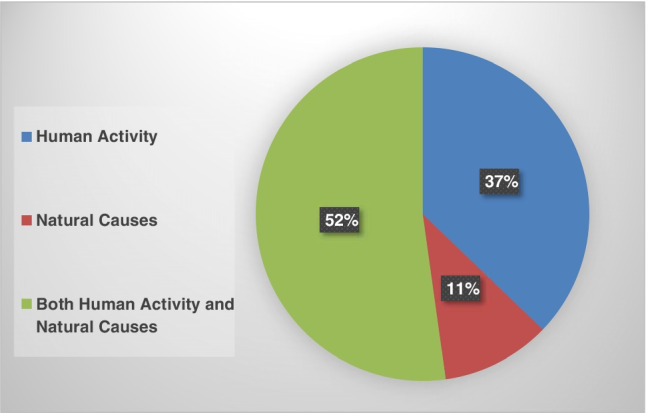
Respondents’ knowledge of climate change main cause

About 48 % and 52 % of the students have chosen the correct climate change definition and main cause, respectively (Figures 5 and 6). The students’ response statistics are presented in Table 5.

**Table 5:** Statistics of respondents’ knowledge of the climate change causes

**Questionaries**		**Responses**	
		Percent	Frequency
What is the best definition for climate change?	The emission of gases into the atmosphere that causes global warming	28.2	297
	Changes that occur in the layers of the atmosphere, such as temperature and precipitation	48.4	509
	Excessive emission of gases	4.8	50
	Studying the human impact on the environment	18.6	196
What is the main reason for climate change, in your opinion?	1-Human activity (burning fossil fuels, increasing the population, increasing industrial activity and the number of cars and gases emitted)	37.2	391
	2-Natural vauses (volcanic eruption and increase in solar activity)	10.6	112
	3-Both human activity and natural causes	52.2	549

### Respondents’ knowledge of the effects of climate change on water resources

Descriptive statistics of the student’s responses are shown in Table 6.

**Table 6:** Descriptive statistics of climate change effects

Questionaries		1	2	3	4	5	Mean	STD.
**Climate change is evident in extreme weather events, and it is actually happening**	**N**	78	99	355	198	322	3.56	1.222
	**%**	7.4	9.4	33.7	18.8	30.6		
**Climate change causes floods**	**N**	162	158	284	164	284	3.24	1.395
	**%**	15.4	15.0	27.0	15.6	27.0		
**Climate change causes droughts**	**N**	59	68	256	250	419	3.86	1.177
	**%**	5.6	6.5	24.3	23.8	39.8		
**Climate change causes less rain and higher temperatures**	**N**	58	58	210	231	495	4.00	1.178
	**%**	5.5	5.5	20.0	22.0	47.1		
**Climate change is causing sea-level rise**	**N**	119	132	343	169	289	3.36	1.308
	**%**	11.3	12.5	32.6	16.1	27.5		
**Climate change negatively affects the amount of water in rivers and groundwater**	**N**	54	73	236	254	435	3.90	1.170
	**%**	5.1	6.9	22.4	24.1	41.3		
**Climate change negatively affects the quality of river water and groundwater**	**N**	100	103	276	229	344	3.58	1.290
	**%**	9.5	9.8	26.2	21.8	32.7		

Analysis of results on the effects of climate change on water resources shows that respondents know how climate change causes less rain and higher temperature. Out of seven questionaries about the climate change effects, respondents ranked “Climate change causes less rain and higher temperatures” as the first important cause. At the same time, apart from the most commonly known effects of climate change on surface water, such as droughts, heatwaves, and water shortages, respondents did not have much knowledge about how climate change affects groundwater quantity and quality. They ranked it as the fourth and sixth, respectively; at the time, studies confirmed a close link with climate change’s adverse effects on groundwater’s quantity and quality (Abdelhalim et al. 2020; Lukač Reberski et al. 2020; Ghazi et al. 2021). The analysis results are presented in Table 7.

**Table 7:** Ranking criteria of the questionaries regarding climate change effects

Climate change effects	RII.	Rank by category	Overall ranking	Relative importance level
Climate change is evident in extreme weather events, and it is actually happening	0.71	5	7	H-M
Climate change causes floods	0.77	3	4	H-M
Climate change causes droughts	0.65	7	9	H-M
Climate change causes less rain and higher temperatures	0.8	1	1	H-M
Climate change is causing sea-level rise	0.78	2	3	H-M
Climate change negatively affects the amount of water in rivers and groundwater	0.71	4	6	H-M
Climate change negatively affects the quality of river water and groundwate**r**	0.67	6	8	H-M

### Students’ knowledge of climate change mitigation measures.

Descriptive statistics of the responses regarding climate change mitigations measures are presented in Table 8.

**Table 8:** Descriptive statistics of climate change mitigations’ measures

Questionaries		1	2	3	4	5	Mean	STD.
The use of modern irrigation techniques reduces the impact of climate change on water resources	**N**	64	107	287	223	371	3.69	1.220
	**%**	6.1	10.2	27.3	21.2	35.3		
The use of modern irrigation techniques reduces the impact of climate change on water resources	**N**	63	79	210	205	495	3.941	1.2278
	**%**	6.0	7.5	20.0	19.5	47.1		

Results of the climate change mitigation measures that would conserve water resources show that the students did not have good information about this matter. The respondents ranked “Increasing awareness of rationalizing water consumption reduces the impact of climate change on water resources” as the first mitigation measure to conserve water, while the agricultural is the first consumer of water in Iraq with 75–80 % of the total water consumption. The analysis results are shown in Table 9.

**Table 9:** Ranking criteria of the questionaries regarding climate change mitigation

Climate change mitigation	RII.	Rank by category	Overall ranking	Relative importance level
Using modern irrigation techniques reduces the impact of climate change on water resources	0.74	2	5	H-M
Increasing awareness of rationalizing water consumption reduces the impact of climate change on water resources	0.79	1	2	H-M

Statistics of the responses for different classifications are presented in Tables 10 and 11. Kendall’s tau and Spearman’s rho correlations were used for assessing the correlation between variables as they do not follow a normal distribution. Table 12 shows the correlation coefficients between respondent majors, stage, and gender with the knowledge of climate change definition and causes. All relationships were positively weak, with the highest Spearman’s rho of 0.15 for the students’ gender with their understanding of climate change main cause by 0.06 for the academic stage versus knowledge of climate change causes.

**Table 10:** Statistics of the responses about the best definition of climate change

Classification		The best definition of climate change				Total
		The emission of gases into the atmosphere that cause global warming	Changes that occur in the layers of the atmosphere, such as temperature and precipitation	Excessive emission of gases	Studying the human impact on the environment	
**Major**	**Medical sciences**	23	83	4	21	131
	**Engineering science**	125	163	22	82	392
	**Applied sciences**	33	26	6	14	79
	**Agriculture and veterinary**	7	21	2	7	37
	**Human Studies**	30	66	5	15	116
	**Literary disciplines**	27	41	5	11	84
	**Artistic disciplines**	0	3	0	0	3
	**Other**	52	106	6	46	210
**Total**		297	509	50	196	1052
**stage**	**First**	64	150	19	46	279
	**Second**	67	153	7	43	270
	**Third**	54	83	4	48	189
	**Fourth**	58	94	9	43	204
	**Fifth**	13	13	5	5	36
	**Sixth**	41	16	6	11	74
**Total**		297	509	50	196	1052
**Gender**	**Female**	154	174	35	97	460
	**Male**	143	335	15	99	592
**Total**		297	509	50	196	1052

**Table 11:** Statistics of the responses about the main cause of climate change

Classification		The main cause of climate change			Total
		Human activity	Natural causes	Both human activity and natural causes	
Major	Medical sciences	44	9	78	131
	**Engineering science**	157	30	205	392
	**Applied sciences**	36	4	39	79
	**Agriculture and veterinary**	13	5	19 (51	37
	**Human Studies**	40	21	55 (47	116
	**Literary disciplines**	25	10	49 (58	84
	**Artistic disciplines**	1	1	1 (33	3
	**Other**	75	32	103 (49	210
**Total**		391	112	549	1052
**Stage**	**First**	99	34	146 (52	279
	**Second**	84	33	153 (57	270
	**Third**	69	24	96 (51	189
	**Fourth**	80	17	107 (52	204
	**Fifth**	18	1	17 (47	36
	**Sixth**	41	3	30 (41	74
**Total**		391	112	549	1052
**Gender**	**Female**	211	43	206 (45	460
	**Male**	180	69	343 (57	592
**Total**		391	112	549	1052

**Table 12:** Correlation coefficients

Category		Correlation Coefficient	
		Kendall’s tau_b	Spearman’s rho
**Respondents’ major**	The best definition of climate change and	−0.005-	−0.006-
	The main cause of climate change and respondents Major	−0.015-	−0.018-
**Respondents’ gender**	The best definition of climate change and respondents Gender	0.010	0.010
	The main cause of climate change and	0.144^**^	0.150**
**Respondents’ stage**	best definition of climate change	−0.053-*	−.063-*
	main cause of climate change	−0.057-*	−0.066-*

*Correlation is significant at the 0.05 level (2-tailed).

**Correlation is significant at the 0.01 level (2-tailed).

**Fig. 7:**
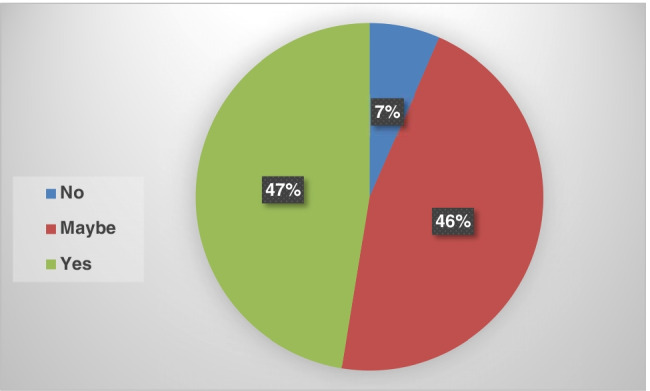
Willingness to participate in supporting climate change initiatives

The final question for the participants was as follows: Do you have a desire to participate in or support initiatives to mitigate and adapt to climate change? The responses are presented in Figure 7, which shows that most students desire to participate and support climate change initiatives. Therefore, it is necessary to think about designing the appropriate programs for them.

There is a dearth of research on college students’ knowledge of climate change (Artz et al. 2012; Bostrom et al. 2012; Wachholz et al. 2014; Parashar et al. 2013; Rideout 2014), while most studies were performed on students at various levels of schooling (Liarakou et al. 2011; Aydin 2010; Bozdogan 2011; Steynor et al. 2020). Most of those researchers approached this problem differently, making each study unique in its method and result.

## Conclusions, recommendations, and policy implications

A true understanding of nature will lead to the emergence of a view that values biodiversity, with the realization that every living being depends on the existence of other creatures. In this study, a questionnaire distributed using Google form in May 2021 was used to assess the undergraduates’ knowledge about climate change effects on water resources. The results showed that the general awareness regarding climate change issues among first and second year students was higher than in the other stages. The general knowledge for all questionaries was between 40 and 50 %. This study is the region’s first research to assess university students’ knowledge and attitudes regarding the impacts of climate change on water resources. The degree of awareness shown by first year students is most likely indicative of the information acquired throughout their school education. In this study, university students were selected as a study sample because they constitute a large and diverse society segment that comes from different social classes. They spend a period ranging between 4 and 6 years in university studies. Therefore, directing and educating this segment will be effectively reflected on the community as they will transmit this information to their homes and families and also in the future to their children.

Integrating the Environmental Education (EE) programs into the Iraqi educational systems as it proposes a systematic educational method through which climate change and other environmental problems may be addressed holistically at all educational levels. This program is ineffectual in the Iraqi educational system, where no formal EE program is implemented at any academic level. Thus, most students acquire knowledge, not through their education but other media, like television, the internet, and social media. These sources are not necessarily trustworthy.

Including environmental and climate change-related themes and academic projects in the curriculum for all stages may help raise awareness engaging students in creating visually appealing activities related to the concerned events. Significant effort must be made to integrate environmental ideas into university curricula to raise environmental consciousness among all students, regardless of academic speciality. This would go a long way toward developing a new generation capable of successfully addressing the problems posed by climate change. Moreover, actively support and encourage global warming remembrance events such as the World Climate Change Day (May 15 ), World Water Day: (22 March), World Desertification Day (17 June), and World Environment Day (5 June) every year by organizing activities and festivals, as well as presentations that convey the required information. Students can also be encouraged to participate in fun activities linked to water conservation and climate change mitigation, such as planting trees and passing out informational flyers.

Finally, more research should be conducted on other society segments such as high school students, farmers, and state employees to indicate the most tenuous one. Then, propose a suitable plan depending on the results.
